# Associations between internet addiction and school engagement among Turkish college students: mediating role of psychological distress

**DOI:** 10.3389/fpsyg.2024.1367462

**Published:** 2024-02-29

**Authors:** Gülçin Güler Öztekin

**Affiliations:** Department of Psychology, Faculty of Science and Letters, Agri Ibrahim Cecen University, Agri, Türkiye

**Keywords:** internet addiction, depression, anxiety, stress, school engagement, college students

## Abstract

**Introduction:**

Internet addiction has become a subject of growing concern with adverse consequences. This study aimed to investigate the mediating effect of psychological distress in the relationship between internet addiction and school engagement.

**Methods:**

Data were obtained from 732 Turkish college students (M = 20.98, SD = 2.58). The data were collected through Young’s Internet Addiction Test, Depression, Stress and Anxiety Scale (DASS21)-Short Form, and University Student Engagement Inventory. Mediation analysis was performed to investigate the mediating effects of depression, anxiety, and stress in the association between internet addiction and school engagement.

**Results:**

The results of the study showed that internet addiction was positively associated with psychological distress and negatively associated with school engagement, whereas psychological distress had a negative relationship with school engagement. This study also found that depression, anxiety, and stress acted as mediators in the relationship between internet addiction and behavioral engagement, emotional engagement, and cognitive engagement.

**Conclusion:**

This study has provided evidence that young adults with tendency of internet addiction may experience school engagement problems as well as psychological problems. Our findings underscore the need for special educational intervention programs aimed at encouraging college youth to limit their internet use for their future due to its negative consequences and raising awareness in this vulnerable group and their families.

## 1 Introduction

College years are a period that supports individuals’ autonomy and affects their success and satisfaction in their future lives. In this period, school engagement emerges as an important factor. School engagement can be defined as the student’s behavioral, emotional, cognitive, and psychological attachment to school. Behavioral engagement includes participation in academic, social, and extracurricular activities. Behaviorally engaged students follow and comply with school rules and norms, participate in the learning process and academic tasks, participate in school-related activities, and do not exhibit disruptive behaviors. Emotional engagement can be defined as the students’ positive and negative reactions to school and its components (teachers, classmates, academics). Emotionally engaged students experience positive emotions such as interest and happiness rather than boredom, sadness, and anxiety. Cognitive engagement encompasses the thoughtfulness and willingness to make the necessary effort in comprehending complex ideas and mastering hard skills. Cognitively engaged students are strategic and self-regulated, have flexibility in problem solving, prefer to work hard, and have positive coping skills in case of failure (Fredricks et al., 2004). These three components seem to be one of the keys to success in academic life.

The growing interest in behavioral, emotional, and cognitive engagement yielded that these were associated with positive academic outcomes such as academic performance (Martínez et al., 2019) and academic achievement (Wang et al., 2019), and serve protective functions in negative situations such as bullying (Forster et al., 2020), academic procrastination (Çıkrıkçı and Erzen, 2020) and college dropout (Bernardo et al., 2022). Participants with low behavioral engagement experienced more difficulties in organizing the study place, time, and materials, complying with school rules, and paying attention in class. Students with low cognitive engagement had difficulty in monitoring their understanding and recognizing the importance of thinking and asking questions about activities in class (Estévez et al., 2021). In addition, high behavioral, emotional, and cognitive engagement contributed to students’ hope, social competence, and well-being (Demirci, 2020). Once engagement to school is established, this contributes to a higher level of investment in the individual’s learning (Syed, 2012). It is clear that this multifaceted concept has a significant impact on individuals in psychological, social and academic areas. Therefore, it is necessary to determine the factors affecting school engagement and find ways to increase behavioral, emotional, and cognitive engagement.

### 1.1 Internet addiction and behavioral, emotional, and cognitive engagement to school

With technological developments, internet has become a resource that individuals frequently apply in the areas of life such as communication, education, and entertainment. With technological devices providing easy accessibility, the internet is an important tool that has advantages such as updating individuals, helping them in their studies, solving problems and establishing good relationships with others. However, a high rate of internet use may have negative consequences (e.g., poor psychological health) (Rayan et al., 2017). For example, Dienlin and Johannes (2022) found that low and excessive use of digital technology was associated with decreased well-being, while moderate use was associated with increased well-being. On the other hand, today’s young people have been involved in these technological changes since the beginning of their lives and with the development of online applications such as online games, online gambling, and social networking sites, technology has become an integral part of their daily lives. This has blurred the distinction between addictive and functional internet use (Young and De Abreu, 2017). Although the internet has become an indispensable resource in our lives, it is important and necessary to make this distinction.

There is still a debate about internet addiction or problematic internet use, but the number of reference sources and studies regarding addictive internet use is increasing. The fifth edition of the Diagnostic and Statistical Manual of Mental Disorders (DSM V) included internet gaming disorder as a condition for further study (APA, 2013). In addition, the neuropsychological and neuroimaging studies regarding the excessive and addictive internet use indicate the association with functional brain changes in some parts of the prefrontal cortex, cortical and subcortical regions. Studies have also provided evidence of structural brain changes. Individuals with internet addiction performed tasks such as measuring cue-reactivity and, some structural changes were observed as well as functional changes in the prefrontal and striatal areas. The results highlight that control processes related to the prefrontal cortex are reduced in internet addicted individuals and this may be related to the loss of control over internet use (Brand et al., 2014). Consistent with these research, in Choi et al. (2014) study, the internet addiction group displayed more impulsivity characteristics than the healthy group, and had higher scores for novelty seeking, which was highly correlated with impulsivity. The internet addiction group also performed worse than the control group on the computerized stop signal test, a test of inhibitory function and impulsivity. Impulsive individuals have low basal dopamine activity and need excitement to increase their arousal levels (Cloninger et al., 1993). These studies have supported that the internet may lead to addiction due to biological changes.

In addition to these studies, some researchers have developed measurement tools and defined some criteria on internet addiction. For example, K. Young (1998) described internet addiction as an impulse control disorder without intoxicant and determined a set of criteria for internet addiction by stating that pathological gambling was most similar to the pathological nature of internet use. These criteria are as follows: Being involved with the internet; increasing need to use the internet to achieve satisfaction; unsuccessful efforts to reduce or stop internet use; mood lability, irritability, or depression with limited internet use; being online for longer than intended; jeopardize the opportunities encountered through internet use; lying to others to hide time spent online and using the internet to escape problems or regulate mood. These criteria shed light on many researchers in determining the degree of internet dependence.

Previous studies conducted with these criteria provided support the negative consequences of internet addiction. For example, internet addiction was associated with sleep problems (Alimoradi et al., 2019), aggressive behaviors (Zhang et al., 2022), suicidality (Yang et al., 2023), loneliness, less social interaction (Yao and Zhong, 2014) as well as impaired physical and mental health (Zhou et al., 2022). It is noteworthy that internet addiction, in addition to these physical, social, and psychological problems, also causes problems in education. This addiction had a negative impact on academic performance (Noreen, 2013), academic achievement (Zhang et al., 2018), academic motivation and school attachment (Demir and Kutlu, 2020). We can understand from these studies how great harm occurs to individuals when internet use turns into addiction.

One of the underlying reasons for the negative effects of such addictions in the field of education may be the impact on school engagement. The expanding literature showed that there was an inverse relationship between behavioral addictions and school engagement. Sampasa-Kanyinga et al. (2019) found that heavy social media use led to lower school connectedness and academic performance among students. A recent study showed that students with high levels of problematic smartphone use spent more time on the smartphone, which reduced the time they devoted to learning and subsequently affected their school engagement and disengagement (Li et al., 2023). In addition, in Rayan et al. (2017) study, 57% of the participants stated that internet had an adverse impact on their educational achievement. These results highlight the negative academic consequences of the problematic internet-technology relationship.

### 1.2 Psychological distress

Psychological distress encompasses depression, anxiety, and stress symptoms. Experiencing intense psychological distress can cause impairment in daily functioning and even lead to common mental disorders (Cuijpers et al., 2009). Additionally, maladaptive psychological functioning in response to stressors and demands represents psychological distress. This distress is characterized by an inability to cope, a change in emotional state and perceived discomfort (Ridner, 2004).

Depression refers to emotional problems that occur with negative emotions and are accompanied by behavioral problems (e.g., withdrawal, inattention) (Tang and Zhang, 2022). Negative consequences associated with depression include impairments in interpersonal, social, occupational, and educational functioning (Thapar et al., 2022). The relevant literature has provided expanding evidence that depression was linked to anxiety, drug use, poor health, criminal behavior, poor social functioning, failure to complete school, and unemployment (Clayborne et al., 2019; Copeland et al., 2021). Anxiety can be defined as the feelings of restlessness and worry triggered by uncertain conditions. Anxiety may be helpful because anxiety supports the search to escape danger. On the other hand, since the nature and place of the threat are uncertain, the person cannot be sure how to act, it becomes more difficult to cope with the uncertain threat, and this psychological situation may be damaging (Zeidner and Matthews, 2010). Previous studies have supported the negative effects of anxiety on a variety of domains, including individuals’ coping style, social support, family functioning, and academic lives (Shao et al., 2020; Gao, 2023). Stress refers to the condition caused by external demands that are uncontrollable, unpredictable and exceed the individual’s regulatory capacity (Koolhaas et al., 2011). Stress affects the individual’s homeostasis. Stressed individuals were more likely to experience low psychological well-being, low school life adjustment and psychosocial morbidity (Kim et al., 2018; Li and Hasson, 2020). Depression, anxiety, and stress may lead to a myriad of psychosocial and academic outcomes which are linked and cause the propagation of difficulties across the lifespan. Thus, determining risk factors, consequences and prevention methods is important for individuals’ quality of life.

### 1.3 Internet addiction, psychological distress, and behavioral, emotional, and cognitive engagement to school

Researchers have determined results that make valuable contributions to psychology by focusing on internet addiction, psychological distress, and school engagement. Fan (2022) revealed the positive relationship between internet addiction and psychological distress. A study showed that the internet addiction group spent more time on the internet with greater lack of control, and found an association between internet addiction and depression (Hasan and Abu Jaber, 2020). Another study revealed that excessive internet use predicted somatic and depressive symptoms, emotional and behavioral problems (Cerruti et al., 2017). In addition, more problematic social media use was found to predict adverse mental health outcomes, such as more depressive and anxiety symptoms (Watson et al., 2022). It is possible to say that internet addiction exacerbates the depression, anxiety, and stress symptoms.

Studies have also demonstrated relationships between psychological distress and decreased school engagement. Higher depressive symptoms led to problems adjusting to the school environment and lower school engagement (Klinck et al., 2020). Vaughn et al. (2011) found that psychological distress and conduct problems were related to behavioral indicators of school disengagement (e, g., absenteeism and cutting class). In addition, there are studies regarding the mediating effect of psychological distress in the literature. In Fekih-Romdhane et al. (2023) study, depression, anxiety and stress acted as mediators in the relationship between three behavioral addictions (Internet, smartphones and Facebook addictions) and schizotypal traits. Anxiety mediated the relationship between smartphone addiction and boredom proneness, loneliness (Malaeb et al., 2022), and between addictive smartphone use and self-esteem (Gao et al., 2021). Depressive and anxiety symptoms showed mediating effects in the relationship between school bullying and academic stress (Chen et al., 2023). These studies have provided evidence that psychological distress may serve as a mediator between behavioral addictions and academic outcomes.

### 1.4 Present study

The latest data of the Turkish Statistical Institute indicated that the internet usage rate was 85.0% in Türkiye in 2022. Unfortunately, the rate of individuals performing online learning activities was 17.1% in 2021 and 15.9% in 2022. Additionally, most of the people using the internet were between the ages of 16–24 and 25–34, which corresponds to their university years (TUIK, 2022). In this period, college students may be at risk of possible behavioral addictions such as internet addiction because of the widespread internet usage. In addition, internet addiction is a comprehensive and serious problem with long-lasting negative consequences. However, it is unclear through which mechanisms internet addiction is associated with school engagement. Previous studies have shown that there is a positive relationship between internet addiction and psychological distress and between psychological distress and school engagement, but no study has been found examining the mediating role of psychological distress in the association of internet addiction on school engagement. To address this gap, we supposed that excessive use of internet, its addictive nature and the associated negative effects may have caused depression, anxiety, and stress, which in turn impede school engagement. To this end, the following hypotheses were generated:

H1: Internet addiction would positively predict depression, anxiety, and stress, and negatively predict behavioral engagement, emotional engagement, and cognitive engagement.

H2: Depression, anxiety, and stress would negatively predict behavioral engagement, emotional engagement, and cognitive engagement.

H3: Depression, anxiety, and stress would have mediating roles in the relationship between internet addiction and behavioral engagement, emotional engagement, and cognitive engagement.

**Figure 1:**
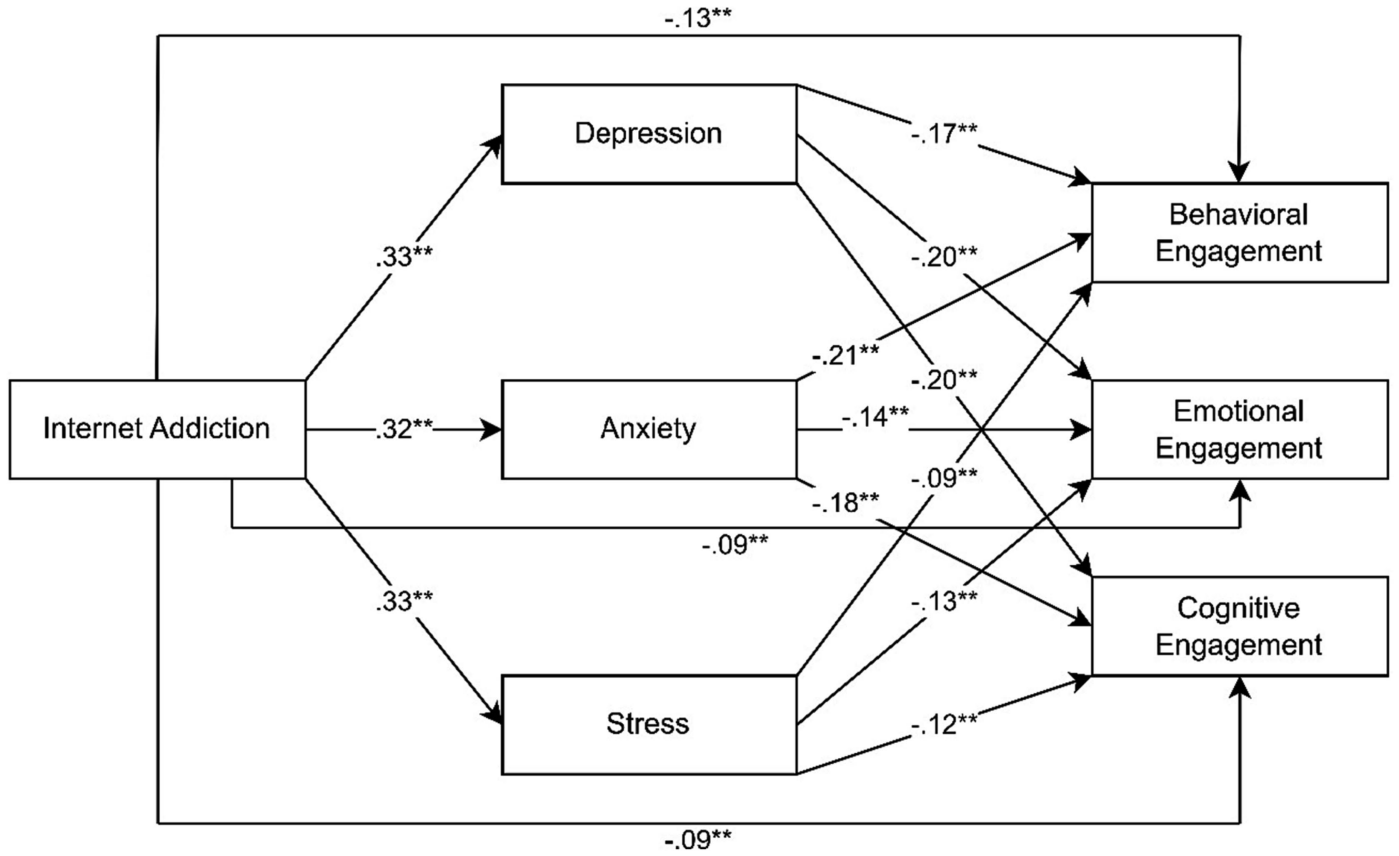
Parallel mediation model showing path coefficients of the proposed model.

The proposed model of the current study is presented in Figure 1.

## 2 Method

### 2.1 Participants

732 college students participated in the study. The age range of the participants was 18 to 43 years, with a mean age of 20.98 years (SD = 2.58). The majority of participants were between the ages of 18–30, and there were six participants between the ages of 30–43. 408 (55.7%) of the participants were female and 324 (44.3%) were male. 184 (25.2%) participants were studying at the Faculty of Education, 263 (35.9%) students were studying at the Faculty of Science and Letters, and 285 (38.9%) students were studying at the Faculty of Health. 285 (38.9%) of the students were freshmen, 170 (23.2%) were sophomores, 128 (17.5%) were juniors, and 149 (20.4%) were senior students. Most of the participants (*n* = 424) had moderate-income families. 194 (26.5%) students were from low-income families, and 114 (15.6%) participants were from high-income families. Most of the students (*n* = 473) were staying in dormitories. 168 (23.0%) students were staying with their families, and 91 (12.4%) students were staying at home (see Table 1). The average internet usage time of college students was 5.30 (SD = 2.99).

**Table 1:** Demographic features of the participants.

Participants	*N*	%
**Gender**		
Female	408	55.7
Male	324	44.3
**Faculty**		
Faculty of Education	184	25.2
Faculty of Science and Letters	263	35.9
Faculty of Health	285	38.9
**Education**		
Freshman	285	38.9
Sophomore	170	23.2
Junior	128	17.5
Senior	149	20.4
**Income**		
High	114	15.6
Moderate	424	57.9
Low	194	26.5
**Place of residence**		
Dormitory	473	64.6
House	91	12.4
Stay with their family	168	23.0
Total	732	100.0

### 2.2 Measures

#### 2.2.1 Young’s internet addiction test

The scale was developed by K. S. Young (1998) and adopted to Turkish culture by Kutlu et al. (2016). The 12-item scale is a unidimensional scale. Each item is rated on a 5-point Likert type ranging from 1 = never to 5 = always. Increasing scores indicate higher levels of internet addiction. Example items are “How often do you stay online longer than you planned?” and “How often do you try to hide how long you are online?.” The Cronbach’s Alpha coefficient of the scale was obtained as 0.85. For this study, the confirmatory factor analysis results were: CMIN: 147.41, df: 50, CFI: 0.95, TLI: 0.94, RMSEA: 0.07, SRMR: 0.03, *p*: 0.00. The Cronbach’s Alpha coefficient was calculated as 0.92.

#### 2.2.2 Depression, Stress and Anxiety Scale (DASS21)-short form

The scale was developed by Lovibond and Lovibond (1995) and adopted to Turkish culture by Yılmaz et al. (2017). The 21-item scale consists of three subscales: depression, anxiety, and stress. Each item is rated on a 4-point Likert-type scale ranging from 0 = not suitable for me to 3 = completely suitable for me. Increasing scores show increased levels of depression, anxiety, and stress. Example items are “I had tremors in my body (for example, in my hands)” and “I could not stand things that distracted me from what I was doing.” The Cronbach’s alpha coefficients were calculated as 0.81, 0.80, and 0.75 for depression, anxiety, and stress, respectively. In this study, the confirmatory factor analysis results were: CMIN: 364.13, df: 186, CFI: 0.98, TLI: 0.98, RMSEA: 0.03, SRMR: 0.02, *p*: 0.00. The Cronbach’s alpha coefficients were 0.92, 0.91, and 0.90.

#### 2.2.3 University student engagement inventory

Maroco et al. (2016) developed the scale and Gün et al. (2019) adapted into Turkish culture. The scale consists of three subscales with 15 items: behavioral engagement, emotional engagement and cognitive engagement. Each item is scored on a 5-point Likert-type scale ranging from 1 = never to 5 = always. High scores show high school engagement. Example items are “I obey the school rules” and “I am happy to be at school.” The Cronbach’s alpha coefficients were found to be 0.66, 0.68 and 0.78 for behavioral engagement, emotional engagement, and cognitive engagement, respectively. In this study, the confirmatory factor analysis results were: CMIN: 350.23, df: 84, CFI: 0.95, TLI: 0.93, RMSEA: 0.06, SRMR: 0.04, *p*: 0.00. The Cronbach’s alpha coefficients were 0.81, 0.80, and 0.84.

### 2.3 Procedure

We created the online survey using Google Form, a secure link for study dissemination. We employed snowball sampling method to collect data. In this method, researchers begin the data collection process with a few participants who meet the research criteria and are invited to become research participants. Participants who agree to take part in the study are then asked to recommend others who meet the research criteria and may be willing to participate in the study, and these new participants continue the same process (Parker et al., 2019). The criteria for inclusion of the study were being at least 18 years old, being a university student, volunteering to participate in the research, and having internet access. We invited the potential participants via social media and sent the link containing the consent form, the aim of the study, their right to withdraw from the survey during or after participation, and the anonymity and confidentiality on the first page, and the measurement package on the other pages. Participants were not allowed to view the questions without agreeing to voluntary participation. The ethics committee of Agri Ibrahim Cecen University approved this study (Ethic Code: 83972).

### 2.4 Data analyses

The normality assumption was tested with skewness and kurtosis scores, with acceptable values between +1.5 and −1.5 (Tabachnick et al., 2013). As a preliminary analysis, mean, standard deviation, Cronbach’s alpha and Pearson correlation coefficients were determined. PROCESS-Macro v4.2 was used to test the mediation with Model 4, and the results were interpreted with unstandardized path estimate (*β*) and squared-multiple correlations (*R^2^*) values (Hayes, 2017). We used 10,000 bootstraps to examine the indirect effects of mediators at 95% confidence intervals. According to Hayes (2017), the confidence intervals, not contain zero indicate that the indirect effect is statistically significant. Both preliminary and mediation analyses were performed using SPSS version 27.

## 3 Results

Preliminary analyses such as means, standard deviations, skewness and kurtosis values, and correlations between variables are presented in Table 2. Skewness values ranged between −0.11 and 0.84, and kurtosis values ranged between −0.05 and −0.69. These results showed that none of the variables violated the normality assumption. Correlation analysis indicated that internet addiction had significant positive correlations with depression (*r* = 0.60, *p* < 0.001), anxiety (*r* = 0.61, *p* < 0.001), and stress (*r* = 0.63, *p* < 0.001), and significant negative correlations with behavioral engagement (*r* = −0.66, *p* < 0.001), emotional engagement (*r* = −0.57, *p* < 0.001), and cognitive engagement (*r* = −0.57, *p* < 0.001). Depression, anxiety, and stress had negative correlations with behavioral engagement (*r* = −0.69, −0.70, −0.67, *p* < 0.001, respectively), emotional engagement (*r* = −0.64, −0.62, −0.62, *p* < 0.001, respectively), and cognitive engagement (*r* = −0.65, −0.64, −0.63, *p* < 0.001, respectively).

**Table 2:** Descriptive statistics, skewness, kurtosis, and correlations.

Variables	M	SD	Skewness	Kurtosis	Correlation						
					1	2	3	4	5	6	7
1. Internet addiction	29.73	9.88	0.42	−0.40	-						
2. Depression	8.19	5.42	0.74	−0.26	0.60**	-					
3. Anxiety	7.11	5.23	0.84	−0.05	0.61**	0.79**	-				
4. Stress	7.84	5.26	0.48	−0.56	0.63**	0.77**	0.79**	-			
5. Behavioral engagement	16.47	4.36	0.01	−0.69	−0.66**	−0.69**	−0.70**	−0.67**	-		
6. Emotional engagement	14.91	4.39	0.36	−0.38	−0.57**	−0.64**	−0.62**	−0.62**	0.70**	-	
7. Cognitive engagement	16.98	4.45	−0.11	−0.61	−0.57**	−0.65**	−0.64**	−0.63**	0.75**	0.66**	-

M, mean; SD, standard deviations; **p < 0.001.

Parallel mediation analyses were performed with a bootstrapping resampling procedure to assess whether depression, anxiety and stress mediated the relationships between internet addiction and behavioral engagement, emotional engagement, and cognitive engagement (see Figure 1). As shown in Table 3, internet addiction significantly predicted depression (*β* = 0.33, *p* < 0.001), anxiety (*β* = 0.32, *p* < 0.001), stress (*β* = −0.33, *p* < 0.001), behavioral engagement (*β* = −0.13, *p* < 0.001), emotional engagement (*β* = −0.09, *p* < 0.001), and cognitive engagement (*β* = −0.09, *p* < 0.001). Internet addiction explained 36% of the variance in depression, 38% of the variance in anxiety and 40% of the variance in stress. Behavioral engagement was significantly predicted by depression (*β* = −0.17, *p* < 0.001), anxiety (*β* = −0.21, *p* < 0.001), and stress (*β* = −0.09, *p* < 0.001). Internet addiction, depression, anxiety, and stress significantly predicted behavioral engagement by explaining 60% of the variance in behavioral engagement. There was a direct effect of internet addiction on behavioral engagement (*β* = −0.13, *p* < 0.001) and internet addiction had indirect effects on behavioral engagement through depression (effect = −0.05, [−0.08, −0.03]), anxiety (effect = −0.06, [−0.09, −0.04]), and stress (effect = −0.03, [−0.06, −0.01]) as presented in Table 4.

**Table 3:** Unstandardized coefficients for the mediation model.

Consequent																												
Antecedent		M_1_ (Depression)					M_2_ (Anxiety)				M_3_ (Stress)						Y_1_ (Behavioral engagement)				Y_2_ (Emotional engagement)				Y_3_ (Cognitive engagement)			
		Coeff.	SE	*t*	*p*		Coeff.	SE	*t*	*p*		Coeff.	SE	*t*	*p*		Coeff.	SE	*t*	*p*	Coeff.	SE	*t*	*p*	Coeff.	SE	*t*	*p*
X (Internet addiction)	a_1_	0.33	0.01	20.63	0.00	a_2_	0.32	0.01	21.29	0.00	a_3_	0.33	0.01	22.15	0.00	c’	−0.13	0.01	−9.48	0.00	−0.09	0.01	−6.01	0.00	−0.09	0.01	−5.70	0.00
M_1_ (Depression)		–	–	–	–		–	–	–	–		–	–	–	–	b_1_	−0.17	0.03	−5.15	0.00	−0.20	0.03	−5.30	0.00	−0.20	0.03	−5.11	0.00
M_2_ (Anxiety)		–	–	–	–		–	–	–	–		–	–	–	–	b_2_	−0.21	0.03	−5.76	0.00	−0.14	0.04	−3.33	0.00	−0.18	0.04	−4.33	0.00
M_3_ (Stress)		–	–	–	–		–	–	–	–		–	–	–	–	b_3_	−0.09	0.03	−2.73	0.00	−0.13	0.04	−3.19	0.00	−0.12	0.04	−3.06	0.00
Constant	i_M1_	−1.71	0.50	−3.38	0.00	i_M2_	−2.64	0.48	−5.47	0.00	i_M3_	−2.19	0.47	−4.59	0.00	i_y_	24.07	0.32	73.25	0.00	21.48	0.37	56.86	0.00	23.57	0.37	62.67	0.00
		*R*^2^ = 0.36					*R*^2^ = 0.38					*R*^2^ = 0.40					*R*^2^ = 0.60				*R*^2^ = 0.48				*R*^2^ = 0.50			
		*F* = 425.95; *p* < 0.001					*F* = 453.50; *p* < 0.001					*F* = 490.93; *p* < 0.001					*F* = 280.33; *p* < 0.001				*F* = 174.27; *p* < 0.001				*F* = 186.09; *p* < 0.001			

X, independent variable; M, mediator variable; Y, dependent variable; Coeff., unstandardized coefficient; SE, standard error.

**Table 4:** Total, direct, and indirect effects.

	Effect	*SE*	BootLLCI	BootULCI
Path 1				
Total effect	−0.29	0.01	−0.31	−0.26
Direct effect	−0.13	0.01	−0.15	−0.10
Total indirect effect	−0.16	0.01	−0.18	−0.14
Internet addiction–>Depression–>Behavioral engagement	−0.05	0.01	−0.08	−0.03
Internet addiction–>Anxiety–>Behavioral engagement	−0.06	0.01	−0.09	−0.04
Internet addiction–>Stress–>Behavioral engagement	−0.03	0.01	−0.06	−0.01
Path 2				
Total effect	−0.25	0.01	−0.28	−0.22
Direct effect	−0.09	0.01	−0.12	−0.06
Total indirect effect	−0.15	0.01	−0.18	−0.13
Internet addiction–>Depression–>Emotional engagement	−0.06	0.01	−0.09	−0.04
Internet addiction–>Anxiety–>Emotional engagement	−0.04	0.01	−0.07	−0.02
Internet addiction–>Stress–>Emotional engagement	−0.04	0.01	−0.07	−0.01
Path 3				
Total effect	−0.25	0.01	−0.28	−0.23
Direct effect	−0.09	0.01	−0.12	−0.05
Total indirect effect	−0.16	0.01	−0.19	−0.14
Internet addiction–>Depression–>Cognitive engagement	−0.06	0.01	−0.09	−0.03
Internet addiction–>Anxiety–> Cognitive engagement	−0.05	0.01	−0.08	−0.03
Internet addiction–>Stress–> Cognitive engagement	−0.04	0.01	−0.07	−0.01

Number of bootstrap samples for percentile bootstrap confidence intervals: 10,000.

Emotional engagement was significantly predicted by depression (*β* = −0.20, *p* < 0.001), anxiety (*β* = −0.14, *p* < 0.001), and stress (*β* = −0.13, *p* < 0.001). Internet addiction, depression, anxiety, and stress significantly predicted emotional engagement by explaining 48% of the variance in emotional engagement. As shown in Table 4, internet addiction had a direct effect on emotional engagement (*β* = −0.09, *p* < 0.001) and indirect effects on emotional engagement through depression (effect = −0.06, [−0.09, −0.04]), anxiety (effect = −0.04, [−0.07, −0.02]), and stress (effect = −0.04, [−0.07, −0.01]).

Cognitive engagement was significantly predicted by depression (*β* = −0.20, *p* < 0.001), anxiety (*β* = −0.18, *p* < 0.001), and stress (*β* = −0.12, *p* < 0.001). Internet addiction, depression, anxiety, and stress significantly predicted cognitive engagement by explaining 50% of the variance in cognitive engagement. As shown in Table 4, internet addiction had a direct effect on emotional engagement (*β* = −0.09, *p* < 0.001) and indirect effects on cognitive engagement through depression (effect = −0.06, [−0.09, −0.03]), anxiety (effect = −0.05, [−0.08, −0.03]), and stress (effect = −0.04, [−0.07, −0.01]). These results indicated that psychological distress partially mediated the relationship between internet addiction and school engagement.

## 4 Discussion

Internet addiction is an important risk factor for individuals. This cause negative effects on social, psychological and biological development, as well as academic and career processes (Demir and Kutlu, 2020). We found that internet addiction was positively associated with depression, anxiety, and stress, and negatively associated with behavioral, emotional, and cognitive engagement which confirmed the first hypothesis of this study. Our results corroborated previous findings in which higher levels of internet addiction were associated with higher depression, anxiety, and stress levels (Akin and Iskender, 2011). Researchers have purported that spending too much time in sedentary pursuits such as internet use poses health risks such as mental health problems (Mougharbel and Goldfield, 2020). Since the excessive use of internet is associated with some social and psychological maladaptive outcomes such as decrease in social interactions, loneliness, low psychological adjustment and life satisfaction (Yıldırım et al., 2023), internet addiction may enhance depression, anxiety and stress. Addictive and prolonged internet use through technological devices can lead to avoidance of real-life social contacts and social isolation, which can trigger depression, anxiety, and stress.

On the other hand, consisted with our findings, a study with Turkish students found that internet addiction predicted school engagement and participants with higher levels of internet addiction experienced less engagement to school (Tas, 2017). In Demir and Kutlu (2020) study, internet addiction negatively affected school engagement which was linked to academic motivation and procrastination. Additionally, college students residing in the dormitory declared higher levels of internet addiction and academic procrastination. Researchers stated that the reasons may be that students living in dormitories have free, easy and cheap access to the internet, and students are more independent and lack parental control, which may increase their desire to overuse the internet (Hayat et al., 2020). Since the internet offers opportunities, such as meeting new people, playing games, sharing, and becoming popular, individuals may turn to virtual environments instead of real social environments and spend a significant amount of time in the virtual environments. This situation may prevent college students from establishing social relationships with their friends and academics and weaken their engagement to school.

The findings of the present study showed that depression, anxiety, and stress negatively predicted school engagement. This relationship indicates that students experiencing depression, anxiety and stress may have difficulty in adapting to the school environment. This notion corresponds to previous findings indicating that psychological distress has an adverse impact on school engagement (Watts et al., 2019). For example, authors demonstrated that the participants who experienced stress scored lower school engagement (Oral, 2020) suggesting that students’ basic psychological needs for relatedness, autonomy, and competence may be an effective starting point for preventing and intervening in stress and its negative relationship with school engagement (Raufelder et al., 2014). Since experiencing depression, anxiety and stress jeopardizes the individuals’ homeostasis, college students may have difficulty engaging in the learning process, experiencing positive emotions towards school, classmates or academics, and self-regulation, and consequently may affect their behavioral, emotional, and cognitive school engagement.

One of the most significant contributions of the current study is clarification in the relationship of internet addiction and school engagement with psychological distress. Our findings showed that psychological distress had a mediating effect in the relationship between internet addiction and behavioral, emotional, and cognitive engagement. In other words, college students with higher internet addiction levels reported more depression, anxiety, and stress symptoms, which in turn led to lower school engagement levels. There are studies in the literature regarding the deleterious effects of behavioral addictions such as depression, anxiety, and stress on individuals’ social and educational lives. For example, Liu et al. (2022) found the indirect effects of problematic internet use on academic engagement through depression. Satici et al. (2023) determined the associations between social media addiction and relationship satisfaction via psychological distress. Internet gaming disorder increased participants’ depression, anxiety and stress levels and subsequently affected their quality of life (Fazeli et al., 2020). Users of internet-related activities may engage excessively in these activities and therefore spend most of their time in these online activities, compromising other important areas of their lives, including their education, family, and social relationships, leading to psychological consequences such as anxiety, depression, and distress (Zaremohzzabieh et al., 2015). Most of the university students participating in this study stay in dormitories where they have easy access to the internet and their internet use is not monitored, and the social activity opportunities of the city they study in are limited. This may have led them to excessive internet use. Social isolation due to the lack of social activity and easy access to many applications over the internet may have put these students in a cycle of depression, anxiety, and stress. Psychological distress may have further permeated their daily moods and further negatively impacted their behavioral, emotional, and cognitive engagement in school.

The current study demonstrates that there are behavioral and psychological problems underlying the school engagement. To successfully engage in school, students need to demonstrate gain awareness in conscious internet use and positive psychological functioning that prepares them to be effective and engaged learners. Therefore, in addition to the obvious benefits of preventing excessive internet use, efforts to achieve psychological adjustment may also affect school engagement outcomes. Furthermore, one of the requirements of the rapidly changing world is employees who can synthesize and evaluate new information, solve problems, and think critically. School engagement and its components is essential for young people to benefit from the advantages of schools and gain the skills they will need to be successful in their future business lives (Fredricks et al., 2004). Therefore, the findings of the present study should alert parents, educators, and government agencies to consider the risks associated with internet addiction on psychological distress and school engagement.

Although our study also extends previous findings, the results of this study should be interpreted within the framework of some limitations. First, the data were self-reported by the participants and may have been subject to bias that could affect the internal validity of the results. Second, this study is a cross-sectional study, and its nature precludes causal inference. Finally, the sample of the study was university students. Therefore, the results can only be generalized to this population.

The findings of this study contribute to the knowledge in the literature on how to support behavioral, emotional, and cognitive commitment to school in college students. The present results also offer several practical implications. This study found that depression, anxiety, and stress levels negatively affected the school engagement levels of internet addicted college students. Thus, reducing the psychological distress levels of students suffering from depression, anxiety and stress may be beneficial to increase their school engagement. More importantly, it becomes imperative for professionals at universities to provide support and information to students by focusing on psychological distress, and this support can help students engagement to the university. In addition, mental health professionals can help students with psychoeducational programs. In particular, the inclusion of content in these programs on rationale internet use, how the internet can make education more effective, psychological distress symptoms, and how to cope with psychological distress can contribute to students’ behavioral, emotional, and cognitive engagement to school. In this regard, these studies carried out by mental health experts at universities can prevent, reduce, or eliminate these factors that affect their engagement to school by raising awareness among students.

## 5 Conclusion

Preventive studies on the development of behavioral addictions, which cause the impairment of the individual’s functionality and inability to adapt due to the abnormal and frequent display of a certain behavior, should be a priority today, as they lead to negative consequences. This study has provided evidence that psychological distress and school engagement were positively and negatively predicted by internet addiction, respectively. Psychological distress negatively affected the components of school engagement among Turkish college students. Behavioral engagement was most susceptible to being influenced by internet addiction and psychological distress, followed by cognitive and emotional engagement, respectively. Depression, anxiety, and stress exacerbated the negative effect of internet addiction on school engagement. Based on the current study results, it is possible to say that reducing internet addiction levels will mitigate psychological distress and foster school engagement. In this regard, given the undeniable impact of school engagement on students’ future, understanding the related factors is pivotal for parents, educators, and government institutions to take effective precautions.

## Data availability statement

The raw data supporting the conclusions of this article will be made available by the authors, without undue reservation.

## Ethics statement

The studies involving humans were approved by the ethical review board of Agri Ibrahim Cecen University, Türkiye. The studies were conducted in accordance with the local legislation and institutional requirements. The participants provided their written informed consent to participate in this study.

## Author contributions

GGÖ: Conceptualization, Data curation, Investigation, Methodology, Validation, Visualization, Writing – original draft, Writing – review & editing, Formal analysis, Software.
